# Visual oscillation effects on dynamic balance control in people with multiple sclerosis

**DOI:** 10.1186/s12984-022-01060-0

**Published:** 2022-08-17

**Authors:** Lara Riem, Scott A. Beardsley, Ahmed Z. Obeidat, Brian D. Schmit

**Affiliations:** 1 Department of Biomedical Engineering, Marquette University and Medical College of Wisconsin, P.O. Box 1881, Milwaukee, WI 53201-1881 USA; 2 Department of Neurology, Medical College of Wisconsin, Milwaukee, WI 53226 USA

**Keywords:** Multiple sclerosis, Virtual reality, Balance, Visual motion, Gait, Object motion, Self-motion

## Abstract

**Background:**

People with multiple sclerosis (PwMS) have balance deficits while ambulating through environments that contain moving objects or visual manipulations to perceived self-motion. However, their ability to parse object from self-movement has not been explored. The purpose of this research was to examine the effect of medial–lateral oscillations of the visual field and of objects within the scene on gait in PwMS and healthy age-matched controls using virtual reality (VR).

**Methods:**

Fourteen PwMS (mean age 49 ± 11 years, functional gait assessment score of 27.8 ± 1.8, and Berg Balance scale score 54.7 ± 1.5) and eleven healthy controls (mean age: 53 ± 12 years) participated in this study. Dynamic balance control was assessed while participants walked on a treadmill at a self-selected speed while wearing a VR headset that projected an immersive forest scene. Visual conditions consisted of (1) no visual manipulations (speed-matched anterior/posterior optical flow), (2) 0.175 m mediolateral translational oscillations of the scene that consisted of low pairing (0.1 and 0.31 Hz) or (3) high pairing (0.15 and 0.465 Hz) frequencies, (4) 5 degree medial–lateral rotational oscillations of virtual trees at a low frequency pairing (0.1 and 0.31 Hz), and (5) a combination of the tree and scene movements in (3) and (4).

**Results:**

We found that both PwMS and controls exhibited greater instability and visuomotor entrainment to simulated mediolateral translation of the visual field (scene) during treadmill walking. This was demonstrated by significant (p < 0.05) increases in mean step width and variability and center of mass sway. Visuomotor entrainment was demonstrated by high coherence between center of mass sway and visual motion (magnitude square coherence = ~ 0.5 to 0.8). Only PwMS exhibited significantly greater instability (higher step width variability and center of mass sway) when objects moved within the scene (i.e., swaying trees).

**Conclusion:**

Results suggest the presence of visual motion processing errors in PwMS that reduced dynamic stability. Specifically, object motion (via tree sway) was not effectively parsed from the observer’s self-motion. Identifying this distinction between visual object motion and self-motion detection in MS provides insight regarding stability control in environments with excessive external movement, such as those encountered in daily life.

## Background

People with MS (PwMS), even those with low disability, have impaired balance during both standing and walking, thereby increasing the risk of falls. In this study, the effects of medial–lateral visual field oscillation and visual object motion on gait variability and balance while treadmill stepping were examined in PwMS. MS has been documented to cause heterogenous deterioration of the sensory systems, manifested in part as slowed conduction of somatosensory pathways [1, 2]. The effects of somatosensory impairment are characterized by decreased foot sensation [3], changes in the relation between muscle strength and gait speed [4], and position sense deficits in the lower limbs [5]. In addition, PwMS have altered temporal-spatial gait patterns [6] as well as increased variability of trunk movement [7] and footfall placement variability [8] during gait, all of which are expected to contribute to the increased likelihood of balance loss and falls [9–12]. To compensate for the impact of the loss in proprioception on balance, PwMS might have greater reliance on the vestibular or visual systems for balance control [13], as documented by a decrease in stability when visual feedback is removed during standing [14–16] and increased sensitivity to visual stimuli during gait [17]. Because of the potential increased importance of vision on gait stability in PwMS, the effects of visual perturbations of different types on body sway and foot placement during gait were examined in this study. Specifically, the effects of oscillation of the entire visual scene and rotation of objects in the scene on balance during treadmill stepping were characterized in PwMS.

Medial–lateral control of dynamic balance is an important aspect of stability during stepping [18] and has been examined through manipulations of visual feedback. The control and movement of the center of mass (CoM) with respect to the base of support is the basis of maintaining upright posture. As the body doesn’t have specific receptors to sense CoM, multisensory integration assists in identifying the current state of the body CoM [19]. Vision is integrated into sensorimotor control of lateral stability during gait [20], suggesting an important role for vision in medial–lateral CoM control. Foot placement, which is related to CoM control, is a critical aspect of maintaining lateral stability during gait [21, 22]. In turn, visual information from the environment is important for foot placement [23–25], and perhaps even more so in PwMS, who might rely more on vision for stability in gait [26].

The sensitivity of CoM sway and foot placement during gait to visual information has previously been examined using medial–lateral oscillations of the visual field to simulate self-motion [27–30]. The response to visual oscillation, referred to as visuomotor entrainment, characterizes the ability to synchronize or adapt motor responses to a visual stimulus [30]. Visual field-of-view oscillations and their resulting effect on sway and foot placement have been used to demonstrate increased reliance on vision with age [31] and MS [8, 17]. Interestingly, the effect of object motion within a scene, which places additional demands on visual processing of movement, has not been studied in PwMS.

Neural processing of object and visual field motion during gait may be disrupted in PwMS. PwMS can demonstrate cognitive decline, including decreased automatic visual processing [32], and slowed processing of visually presented objects [33]. Declines in information processing speed likely impact motor function, as PwMS decrease gait speed when simultaneously performing cognitive tasks (see review by [34, 35]). Further, PwMS have smaller increases in functional near-infrared spectroscopy signals in the prefrontal cortex during dual-task walking conditions, consistent with decreased attention capacity [36]. The relative effects of object motion and visual field-of-view movement during gait have not been examined in MS, despite evidence suggesting a link between object motion and imbalance during ambulation. While motion of the entire visual field and object motion share similar visual processing pathways in the brain, they engage different intermediate processing centers [37]. Within MS, symptoms associated with disrupted object motion perception have been observed. This includes, for example, the interpretation of object movement when there is absence of such (oscillopsia) [38] and disrupted contrast perception [39] associated with perceiving motion from form [40]. The effect of object motion on movement perception could influence sway during gait, akin to the visuomotor entrainment observed with visual field oscillation [30, 31]; these effects could be altered in PwMS due to adaptations in visual processing.

The present study examined the effect of medial–lateral oscillations of the entire visual field of view (referred to as scene oscillations to simulate self-motion) and of objects within the scene (swaying oscillations of virtual trees) on treadmill stepping in PwMS and healthy age-matched controls using virtual reality (VR). To quantify the visuomotor effects of visual motion on gait, CoM and foot placement were measured. We hypothesized that PwMS are unable to properly parse object movement from self-movement, causing a disruption in postural sway during ambulation when objects move independently in the visual scene. Specifically, we expected to see increases in step width (and variability), peak to peak center of mass (and variability) and change in medial lateral step placement in response to challenging balance conditions from visual field oscillation. However, we expected this to be present more in PwMS than controls, and prevalent in conditions with object motion in PwMS. Thus, our intent was to demonstrate the importance of visual object motion on dynamic balance control in PwMS.

## Methods

This study examined the effect of visually simulated self-motion (scene oscillation) and object motion (tree sway) on stepping variability, foot placement, and CoM motion in PwMS. Visual manipulations were applied to participants with MS and healthy age-matched controls while stepping on a treadmill wearing a head mounted virtual reality (VR) display. Medial–lateral visual oscillations of the entire scene and sway of the virtual trees were used to examine the effect of simulated self and object motion respectively. CoM and characteristics of the base of support (i.e., foot placement) were measured to characterize dynamic stability and response to the visual stimulus.

### Participants

Fourteen participants with MS (clinically definite MS diagnosed by neurologist, mean ± standard deviation, age: 49 ± 11 years, mass: 73.1 ± 13.4 kg, 6 female) and 11 healthy adults with a similar distribution of age (age: 53 ± 12 years, mass: 70.5 ± 11 kg, 6 female), were recruited by word of mouth and completed the study. The inclusion criteria for MS participants were as follows: the ability to stand and walk independently without aid or an orthosis for more than 8 min (self-reported at screening), and a Functional Gait Assessment (FGA) and Berg Balance Scale (BBS) score greater than 15 and 28, respectively. Participants were excluded if they had vision impairments (e.g., double vision, blurred vision, etc.), pain that interfered with their ability to independently maintain balance for at least 8 min, orthopedic or neurological deficiencies (e.g., recent orthopedic surgeries or inability to cognitively understand instructions), or recent (within twelve months) lower extremity surgeries. To characterize dynamic balance in each participant, self-selected gait speed was measured using the 10 m walk test, postural balance was assessed using the BBS and dynamic balance (postural stability while walking) was assessed using the FGA (Table 1). The study was approved by the Institutional Review Board at Marquette University, Milwaukee, WI, and all participants provided written informed consent prior to participation.

**Table 1:** Participant characteristics; average and ± 1 standard deviation are shown

	Walking speed (m/s)	FGA (out of 30)	BBS (out of 56)	10 meter walking (s) (Normal, Fast)	
Control (*n* = 11)	0.90 ± 0.10	30.0 ± 0.0	56.0 ± 0.0	5.34 ± 0.70	3.89 ± 0.41
MS (*n* = 14)	0.74 ± 0.17	27.8 ± 1.8	54.7 ± 1.5	6.70 ± 0.9	5.00 ± 1.15
Statistical Analysis	p < 0.05 t = 2.75	p < 0.01 t = 3.96	p < 0.01 t = 2.85	p < 0.01 t = − 4.13	p < 0.01 t = − 3.05

The results of unpaired t-tests between the two groups are shown, in which the p-value (p) and t-value (t) are provided. The degrees of freedom was 23 for all tests. Note: the walking speed was the actual speed used on the treadmill for experimentation, while the 10-m walking time was used as an assessment of gait function. For the FGA, in older adults scores < 22 would indicate a risk for falls with ~ 4 points indicating a minimal clinically important difference [62]. For the BBS a value of < 40 would indicate an increased fall risk [63]. For the 10-m walking test the minimal detectable change is 1.56 s [64]

### Experimental protocol

**Fig. 1:**
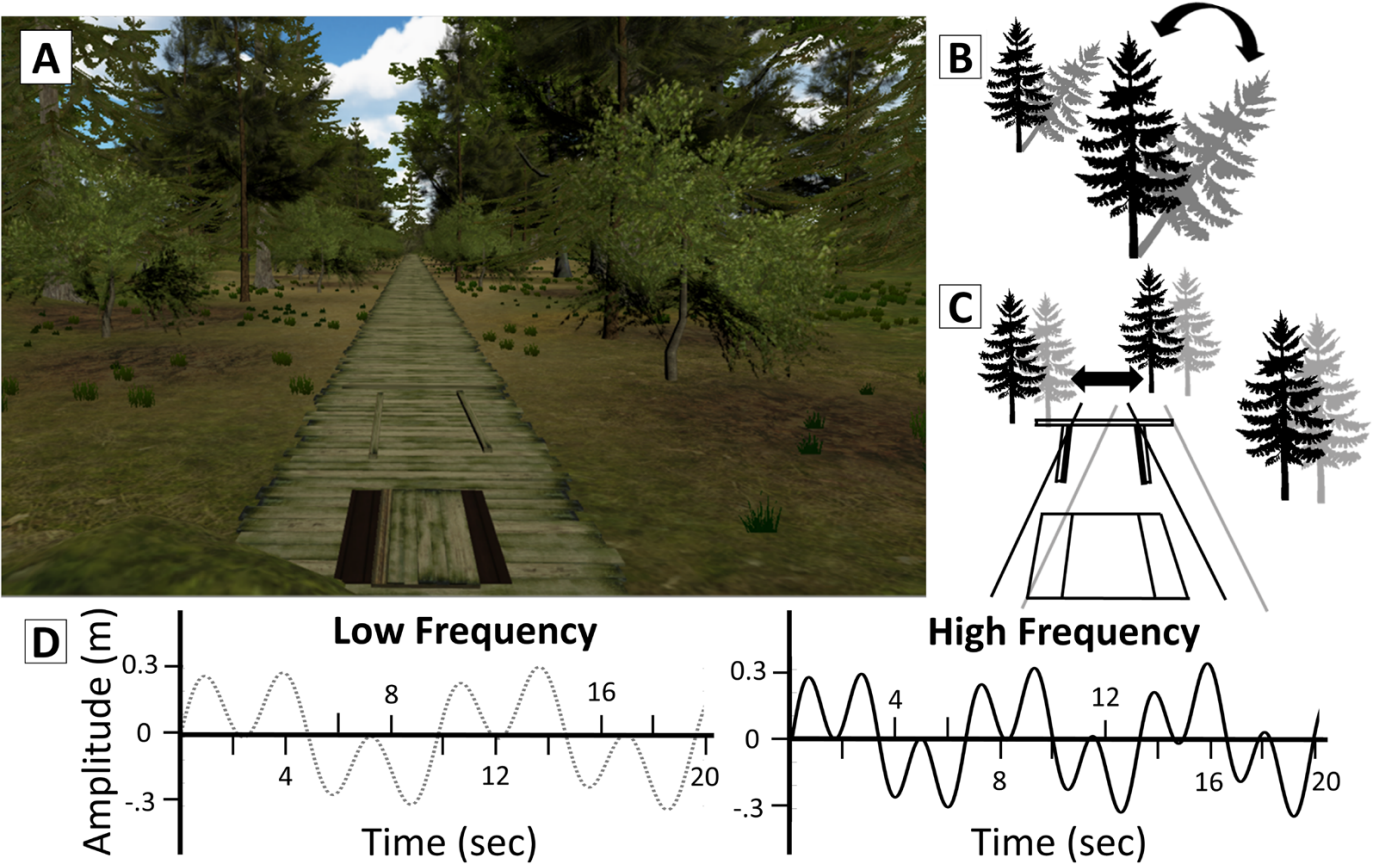
Overview of the experimental setup. **A** The virtual environment presented during the experiment. **B** Object perturbation, in which the tree swayed by rotating from the base. **C** Scene perturbation, in which the entire virtual environment translated left and right. **D** The profile of the perturbation using the low frequency pairing (left) and the high frequency pairing (right) of two sinusoids

During the experiment, participants performed a series of walking tasks designed to characterize the contribution of visual feedback to balance control of gait using an immersive virtual environment. During testing, participants walked on a Woodway split belt treadmill (Woodway USA, Waukesha, WI). Prior to testing, participants completed a warmup on the treadmill to determine their self-selected treadmill speed (Table 1). When participants were comfortable with the setup and task (after an 8-min warm-up), they completed 2.5 min of normal stepping without the VR headset to provide a baseline characterization of gait. After a 2-min break, participants donned an HTC Vive head-mounted display (HMD) (HTC, Taoyuan City, Taiwan). The HMD provides an immersive virtual environment within a 107° horizontal and 107° vertical field of view using two 1080 × 1200 pixel displays (one per eye). The VR headset was used to present an immersive virtual environment for the remaining test conditions. The virtual environment (shown in Fig. 1A) consisted of a wooden bridge within a forest scene created in Unity (Unity Technologies, San Francisco, CA) and housed within the SteamVR plugin, with some elements created within Blender (Blender Foundation, Netherlands). During all VR conditions, the forward movement through the scene was coupled to the treadmill speed to prevent tripping. The frame rate was kept at 90 frames per second, the Vive’s upper limit, to reduce potential nausea. As participants walked, their movement in space was tracked in real time using two Vive lighthouse cameras to account for visual flow changes based on head position, rotation, and body orientation. For safety, the virtual bridge included handrails mapped to the physical handrails of the treadmill and participants wore a safety harness that connected loosely to a support frame above and in front of the participant. After an additional habituation period to allow the participant to become comfortable walking in the VR environment, baseline gait was recorded for 2.5 min of normal walking while participants wore the VR headset (‘Normal’ condition).

The VR headset was used to create ‘perturbations’ of the visual field while stepping on the treadmill. These virtual environment perturbations consisted of scene perturbations, simulating changes in visually perceived self-motion through the scene, and object perturbations simulating independent object motion within the environment. Scene perturbations consisted of medial–lateral translations of the entire virtual environment (Fig. 1B) and object perturbations entailed swaying of virtual trees about their base (Fig. 1C). The perturbation profile was an oscillatory pattern that consisted of the sum of two sine waves (a slower and a faster driving frequency) at either a low pair of frequencies (0.10 and 0.31 Hz) or a relative high pair of frequencies (0.15 and 0.465 Hz) (Fig. 1D). The total sum of sine wave amplitude was 0.175 m for scene perturbations and 5 degrees for object perturbations. The perturbation amplitude and frequency were chosen based on prior research using medial–lateral visual perturbations that found 0.175 m to significantly affect older adults’ lateral foot placement [31]. Five degrees was chosen to provide 0.175 m circumferential distance traveled by the tree at eye level. Tree sway was used instead of translation as sway of the tree object was more natural and more closely resembled what might be encountered naturally while ambulating through a real environment.

Following baseline measurements, participants completed four experimental conditions, 2.5 min each, while walking with the VR headset. The order of the four experimental conditions were randomized across participants and included: (1) virtual environment scene perturbations at low frequency pairing (‘Scene Low’ condition), (2) virtual environment scene perturbations at high frequency pairing (‘Scene High’ condition), (3) object perturbations at low frequency pairing (‘Tree Low’ condition), and (4) combined virtual environment scene perturbations at high frequency pairing and object perturbations at low frequency pairing (‘Combined’ condition). We tested both frequency pairings of the scene perturbation to analyze the effect of scene frequency on gait, to allow comparison of object and scene perturbations at the same frequency, and to combine the object and scene perturbation in the ‘Combined’ condition without overlapping frequencies between scene and object motion perturbations. At the end of the experiment, a simulator sickness survey (SSQ) [41] was administered to ensure that no sickness was produced by the virtual reality environment (Table 2).

**Table 2:** Effect of the virtual reality environment on simulator sickness

Simulator sickness		
Composite score (0 = None; 3 = Severe)	Control (*n* = 11)	MS (*n* = 14)
Oculomotor	0.1 ± 0.3	0.2 ± 0.2
Nausea	0.2 ± 0.3	0.2 ± 0.2

As participants walked on the treadmill, gait kinematics were measured at 120 Hz using a 14-camera Optitrack Flex-13 motion capture system (Natural Point, Corvallis, OR) with a 21–marker modified Helen-Hayes marker model. Four infrared markers were placed on the upper body (the C7, the clavicle, and the right and left acromion). Five markers were placed on the pelvis (right and left anterior superior iliac spine, right and left greater trochanter, and the sacrum), and twelve additional markers were placed on the right and left medial knee, lateral knee, medial ankle malleoli, lateral ankle malleoli, second metatarsal head and fifth metatarsal head. Six rigid clusters, each consisting of three markers organized in an equilateral triangle, were placed on the right and left thigh, shank, and heel.

### Data analysis

Anatomical markers from the motion capture system were labeled in AMASS (C-Motion, Germantown, MD) and kinematic signal processing was completed in Visual3D (C-Motion). Subsequent data analysis was performed in MATLAB (MathWorks, Natick, MA). Gait events were identified using the z-position (vertical) data of the superior heel marker from the foot cluster, together with its sagittal plane marker trajectory, and checked for consistency with the kinematic model as described by Zeni et al. [42]. We analyzed a minimum of 150 steps for each 2.5-min trial.

Participants’ dynamic responses to visual perturbations were characterized via two analyses. First, coherence was calculated as a measure of how closely postural sway followed the visual perturbations. The medial–lateral center-of-mass (CoM) was estimated using three pelvic markers (Eq. 1),

1 $$\mathrm{CoM}=\mathrm{SACR}+0.105\times [(\mathrm{RHIP}\_\mathrm{JC }+\mathrm{ LHIP}\_\mathrm{JC})/2-\mathrm{SACR}]$$

where SACR is the medial–lateral position of the sacrum marker, LHIP_JC and RHIP_JC are the left and right hip joint centers in the medial–lateral direction found using Visual3D, and 0.105 is a constant corresponding to the proximal distance from the hip joint to midpoint of the pelvis [43]. To quantify coherence, we used a method similar to Logan and colleagues [44]; first the Fourier transform of the visual perturbation profile and the demeaned medial–lateral CoM movement were calculated for the first 2 min of each experimental condition. Next, one-sided power spectral densities and cross-spectral densities (CSDs) were calculated using Welch’s method with a 20-s Hanning window and one-half interval overlap. Complex coherence (${C}_{vc}(f)$) was calculated as shown in Eq. 2,

2 $${C}_{vc}(f)={P}_{vc}\left(\mathrm{f}\right)/ \sqrt{{P}_{vv}(f){P}_{cc}(f)}$$

where ${P}_{vc}\left(f\right)$ is the CSD of the visual perturbation (*v*) and the CoM movement (*c*), ${P}_{vv}(f)$ and ${P}_{cc}(f)$ are the autospectral densities of the visual perturbation and CoM movement signals, respectively. We then found the magnitude squared coherence and phase from the ${C}_{cv}(f)$ and averaged the values across all participants within each group for each condition.

**Fig. 2:**
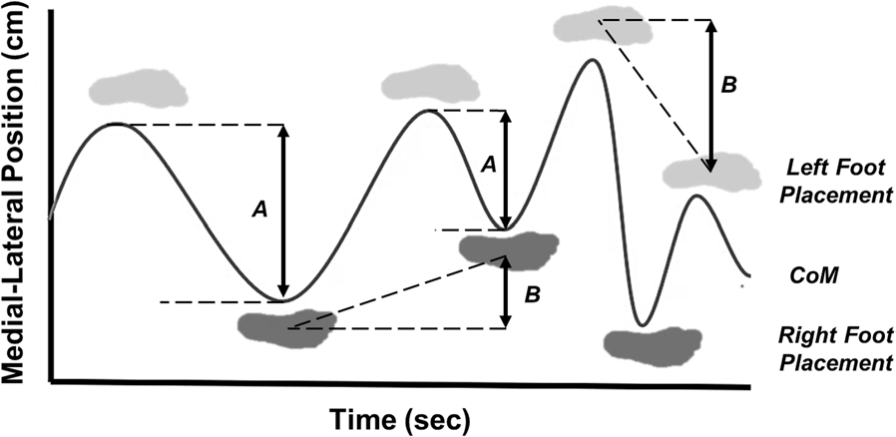
Overview of the change in medial–lateral foot placement and peak to peak center of mass measurements. The graphic depicts the medial lateral position (cm) of CoM (black line) as related to left (light grey foot) and right (dark grey foot) foot placement over time (s). **A** Example peak to peak CoM measurements. **B** Example change in medial lateral foot placement measurements

Second, we quantified the kinematics of the response to visual perturbations using calculations of step width (SW) and CoM motion. SW was calculated using the position of the lateral ankle markers at heel strike. SW was the medial–lateral distance between the right and left marker positions and was normalized to the participant’s leg length. The mean and standard deviation of SW were calculated over the gait cycles of each condition, for each participant. Next, change in medial lateral foot placement was obtained by finding the absolute change in medial–lateral ankle position with respect to each foot from successive heel strikes. Lastly, we quantified CoM movement by measuring the peak-to-peak distance in medial–lateral center of mass over each gait cycle and calculating the mean and standard deviation of this distance for each condition and participant. Examples are shown in Fig. 2.

### Statistical analysis

For statistical analysis, coherence (magnitude squared and phase) values at the slower (0.1 or 0.15 Hz) and higher (0.3 or 0.465 Hz) driving frequencies were used. Separate two-way mixed repeated-measures ANOVAs were performed for the slower driving and higher driving frequencies. In each ANOVA, Group (MS, Control) was specified as a between-subject factor and visual stimulus as a within-subject factor (Coherence Stimulus Condition). The Coherence Stimulus Condition at the slower frequencies included: Tree Low condition at 0.1 Hz (response to object motion), Combined condition at 0.1 Hz (response to object motion while exposed to simulated self-motion), Scene Low condition at 0.1 Hz (response to slower moving simulated self-motion), Scene High condition at 0.15 Hz (response to faster moving simulated self-motion), and Combined condition at 0.15 Hz (response to faster moving simulated self-motion in the presence of object motion). The Coherence Stimulus Condition for higher driving frequencies included: Tree Low condition at 0.31 Hz, Combined (Tree) condition at 0.31 Hz, Scene Low condition at 0.31 Hz, Scene High condition at 0.465 Hz, and Combined (Scene) condition at 0.465 Hz.

Gait data were normally distributed as determined by Shapiro–Wilk tests. Separate two-way mixed repeated-measures ANOVAs were also performed for average SW, average peak-to-peak CoM, variability in SW, variability in peak-to-peak CoM, and change in medial–lateral foot placement. Experimental conditions (‘Normal’, ‘Tree Low’, ‘Scene Low’, ‘Scene High’, ‘Combined’) were specified as within-subject factors and Group (MS, Control) was the between-subject factor in the analyses. Post-hoc Tukey’s pairs comparisons with respect to the ‘Normal’ condition were conducted when a significant ANOVA effect was observed. p < 0.05 was considered significant for all tests.

## Results

**Fig. 3:**
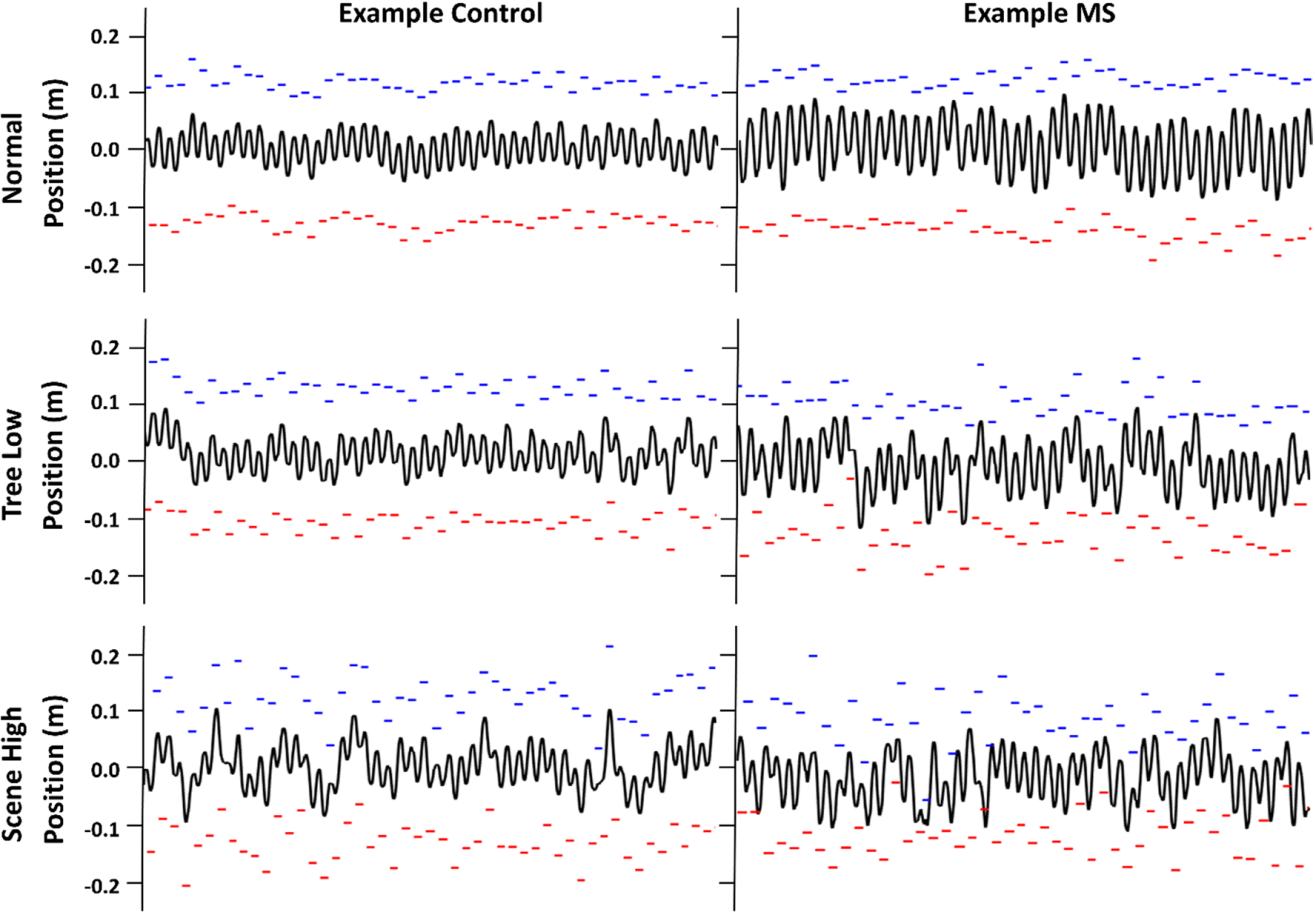
Example medial–lateral position of center of mass and foot placement over a single trial (150 s) for a typical control (left column) and MS (right column) participant. The black line represents the center of mass position, and the red and blue marks represent right and left foot placements from heel strike to toe off respectively. The conditions shown are for the ‘Normal’ condition (top row), ‘Tree Low’ condition (middle row), and ‘Scene High” condition (bottom row)

The results of the study are highlighted in Table 3 below, with the mean and one standard deviation for each group and condition shown, with statistically significant results highlighted. The results of this study consisted of two main findings. (1) Both PwMS and age matched controls exhibited entrainment of the CoM to visual oscillations of the scene or oscillations of objects within the scene. This observation was evidenced by high coherence at the visual movement frequencies as well as significant changes to CoM and SW. (2) PwMS exhibited significantly greater variability and changes to stepping kinematics with object oscillations compared to controls. This is demonstrated in the group results in Table 3, and an example in Fig. 3, in which a typical control and MS participant’s CoM and foot position is displayed across scene and object oscillation conditions. As shown, both the MS and control participant demonstrated an increased variability in stepping kinematics compared to the ‘Normal’ condition for scene perturbations (Table 3 and in the example in Fig. 3); however, the participant with MS also demonstrated increased variability in the object perturbation condition, while the control variability was lower. Both groups had similar variability at the scene high condition. Note that these results appeared without evidence of simulator sickness as measured using the SSQ (Table 2).

**Table 3:** Overview of the results

Group results: magnitude squared coherence and phase					
	Group	Magnitude squared coherence (*low frequency*)	Phase in radians (*low frequency*)	Magnitude squared coherence (*high frequency*)	Phase in radians (*high frequency*)
Tree Low	Control	0.20 ± 0.17	0.66 ± 1.04	0.52 ± 0.21	1.37 ± 0.67
	MS	0.24 ± 0.11	0.41 ± 0.85	0.40 ± 0.20	1.65 ± 0.74
Scene Low	Control	0.42 ± 0.19	2.23 ± 1.66	0.87 ± 0.11	− 2.48 ± 0.46
	MS	0.44 ± 0.26	0.56 ± 2.44	0.73 ± 0.29	− 1.49 ± 1.45
Scene High	Control	0.58 ± 0.27	− 0.36 ± 2.96	0.86 ± 0.20	− 1.71 ± 0.59
	MS	0.60 ± 0.22	0.83 ± 2.68	0.80 ± 0.23	− 1.28 ± 0.93
Combined—Tree Component	Control	0.18 ± 0.19	0.12 ± 1.59	0.85 ± 0.15	1.14 ± 1.55
	MS	0.18 ± 0.13	0.62 ± 0.78	0.72 ± 0.25	0.98 ± 1.48
Combined—Scene Component	Control	0.58 ± 0.26	1.21 ± 2.68	0.26 ± 0.19	− 1.46 ± 0.46
	MS	0.50 ± 0.21	− 0.39 ± 2.81	0.28 ± 0.23	− 1.56 ± 0.65

Significant group statistics: magnitude squared coherence and phase				
Group	p-Value	F-Value	Degrees of Freedom	${\eta }_{p}^{2}$
Magnitude Squared Coherence of Low Frequency Pairing: Main Effect—Coherence Stimulus Condition	< 0.001	15.36	4.20	0.75
Magnitude Squared Coherence of High Frequency Pairing: Main Effect—Coherence Stimulus Condition	< 0.001	38.778	4.20	0.89
Phase of Coherence of High Frequency Pairing: Main Effect—Coherence Stimulus Condition	< 0.001	131.79	4.20	0.96

Group results: stepping characteristics						
Condition	Group	Step width (Normalized)	Step width variability (Normalized)	Peak to peak COM (meters)	Variability peak to peak COM (meters)	ML foot placement (meters)
Normal	Control	0.21 ± 0.03	0.027 ± 0.007	0.054 ± 0.01	0.010 ± 0.003	0.016 ± 0.00
	MS	0.27 ± 0.05	0.031 ± 0.008	0.076 ± 0.02	0.012 ± 0.003	0.019 ± 0.01
Tree Low	Control	0.21 ± 0.02	0.026 ± 0.005	0.055 ± 0.01	0.011 ± 0.003	0.018 ± 0.01
	MS	0.27 ± 0.04	0.039 ± 0.011	0.082 ± 0.02	0.017 ± 0.004	0.031 ± 0.01
Scene Low	Control	0.22 ± 0.03	0.043 ± 0.135	0.073 ± 0.01	0.022 ± 0.007	0.049 ± 0.02
	MS	0.30 ± 0.06	0.048 ± 0.017	0.093 ± 0.02	0.025 ± 0.008	0.051 ± 0.02
Scene High	Control	0.23 ± 0.03	0.056 ± 0.013	0.070 ± 0.01	0.022 ± 0.004	0.054 ± 0.01
	MS	0.30 ± 0.05	0.055 ± 0.018	0.092 ± 0.02	0.025 ± 0.006	0.072 ± 0.04
Combined	Control	0.23 ± 0.03	0.057 ± 0.220	0.068 ± 0.01	0.018 ± 0.004	0.053 ± 0.02
	MS	0.29 ± 0.05	0.057 ± 0.019	0.089 ± 0.02	0.024 ± 0.006	0.043 ± 0.02

Significant group statistics: magnitude squared coherence and phase				
Group	p-value	F-value or *t-value	Degrees of freedom	${\eta }_{p}^{2}$ or *Cohen’s d
Step Width: Main Effect from Normal—Scene Low	< 0.01	8.89	1.23	0.28
Step Width: Main Effect from Normal—Scene High	< 0.01	12.64	1.23	0.36
Step Width: Main Effect from Normal—Combined	< 0.05	7.05	1.23	0.24
Variance in Step Width: Main Effect from Normal—Scene Low	< 0.001	44.35	1.23	0.66
Variance in Step Width: Main Effect from Normal—Scene High	< 0.001	94.42	1.23	0.80
Variance in Step Width: Main Effect from Normal—Combined	< 0.001	67.54	1.23	0.75
Variance in Step Width: Interaction Effect from Normal—Tree Low	< 0.05	6.99	1.23	0.23
Variance in Step Width: Interaction Effect from Normal—Tree Low Post Hoc between MS vs Controls in Tree Low	< 0.01	*− 3.66	18.14	*− 1.35
Variance in Step Width: Interaction Effect from Normal—Tree Low Post Hoc in MS between Normal and Tree Low	< 0.01	*− 3.36	13	*− 0.79
Peak to Peak CoM: Main Effect from Normal—Scene Low	< 0.001	23.21	1.23	0.50
Peak to Peak CoM: Main Effect from Normal—Scene High	< 0.001	37.58	1.23	0.62
Peak to Peak CoM: Main Effect from Normal—Scene Combined	< 0.001	17.26	1.23	0.43
Variance in Peak to Peak CoM: Main Effect from Normal—Scene Low	< 0.001	59.64	1.23	0.72
Variance in Peak to Peak CoM: Main Effect from Normal—Scene Low	< 0.001	140.16	1.23	0.86
Variance in Peak to Peak CoM: Main Effect from Normal—Scene Low	< 0.001	78.94	1.23	0.77
Variance in Peak to Peak CoM: Interaction Effect from Normal—Tree Low	< 0.01	10.78	1.23	0.32
Variance in Peak to Peak CoM: Interaction Effect from Normal—Tree Low Post Hoc between MS vs Controls in Tree Low	< 0.01	*− 4.22	23	*− 1.70
Variance in Peak to Peak CoM: Interaction Effect from Normal—Tree Low Post Hoc in MS between Normal and Tree Low	< 0.01	*− 4.47	13	*− 1.33
Change in Medial Lateral Foot Placement: Main Effect from Normal—Scene Low	< 0.001	59.12	1.23	0.72
Change in Medial Lateral Foot Placement: Main Effect from Normal—Scene Low	< 0.001	49.39	1.23	0.68
Change in Medial Lateral Foot Placement: Main Effect from Normal—Scene Low	< 0.001	60.00	1.23	0.73
Change in Medial Lateral Foot Placement: Interaction Effect from Normal—Tree Low	< 0.01	9.93	1.23	0.30
Change in Medial Lateral Foot Placement: Interaction Effect from Normal—Tree Low Post Hoc between MS vs Controls in Tree Low	< 0.01	*− 3.31	13	*− 1.22
Change in Medial Lateral Foot Placement: Interaction Effect from Normal—Tree Low Post Hoc in MS between Normal and Tree Low	< 0.01	*− 4.42	13	*− 1.07

The top portion of the table displays the mean and standard deviation for each group and condition. The bottom portion of the table displays the significant results. ${\eta }_{p}^{2}$: partial eta squared

### Evidence of visuomotor entrainment using coherence analysis

**Fig. 4:**
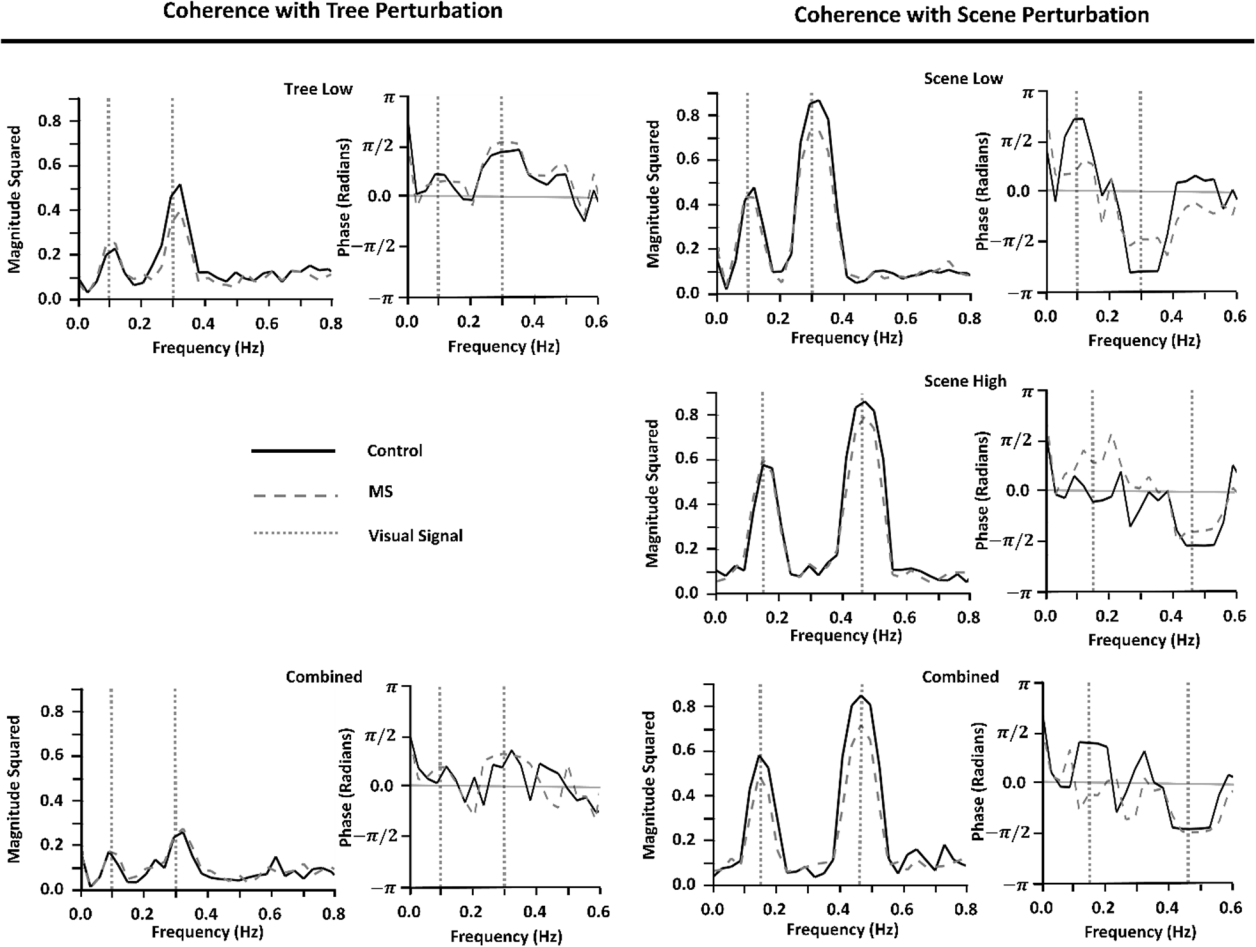
The magnitude squared (1st and 3rd column) and phase (2nd and 4th column) of the coherence between perturbation and CoM movement as a function of frequency (Hz) averaged across MS (grey dashed) and control (black) participants. The visual perturbation driving frequencies are shown by the dotted grey vertical lines for the object (tree, left) and scene (right). Results are shown for each of the Coherence Stimulus Conditions (‘Tree Low’, ‘Scene Low’, ‘Scene High’, ‘Combined’). A significant main effect of Coherence Stimulus Condition was found and is presented in the Results

The coherence between CoM sway and visual oscillations indicated that visuomotor entrainment was achieved, in which frequency of CoM sway was altered to that of the visual perturbations. Further, the results demonstrated that the magnitude squared coherence was dependent on the visual stimulus (object compared to simulated self-motion), but not the group (MS vs controls). As shown in Fig. 4, high magnitude squared coherence and the phase values between the visual perturbation and medial–lateral position of the CoM increased at the visual perturbation driving frequencies in both groups. When the visual perturbation was applied to the scene, higher magnitude squared coherence values were observed with scene perturbations (~ 0.8) compared to object perturbations (~ 0.3). The two-way repeated measure ANOVA for the magnitude squared coherence of the low frequency pairing demonstrated a significant main effect of Coherence Stimulus Condition (p < 0.001; F = 15.36; df = 4.20, partial eta squared (${\eta }_{p}^{2}$) = 0.75). No other significant effects were observed for the interaction of Coherence Stimulus Condition × Group (p = 0.702; F = 0.549; df = 4.20, ${\eta }_{p}^{2}$= 0.10) and between-subject effects (p = 0.991; F < 0.001 df = 1.23, ${\eta }_{p}^{2}$< 0.01). Subsequent post-hoc analyses revealed a significant difference between the Tree Low condition and Scene Low, Scene High, and Combined (with respect to the scene) conditions (p < 0.05). Similarly, there was a significant difference between the Combined condition (with respect to the tree) and Scene Low, Scene High, and Combined (scene) conditions (p < 0.05). No other significant differences between conditions were found. The two-way repeated measure ANOVA for the magnitude squared coherence of the high frequency pairing demonstrated a significant main effect of Coherence Stimulus Condition (p < 0.001; F = 38.778; df = 4.20, ${\eta }_{p}^{2}$= 0.89). No other significant effects were observed for the interaction of Coherence Stimulus Condition × Group (p = 0.550; F = 0.782; df = 4.20, ${\eta }_{p}^{2}$= 0.14) and between-subject effects (p = 0.151; F = 2.209; df = 1.23, ${\eta }_{p}^{2}$= 0.09). Subsequent post hoc analyses revealed a significant difference between the Tree Low condition and Scene Low, Scene High, Combined (with respect to the tree) and Combined (with respect to the scene) conditions (p < 0.05). A significant difference between the Combined (with respect to the tree) and Tree Low, Scene Low, Scene High, and Combined (with respect to the scene) conditions was also observed (p < 0.05).

The results showed a leading phase in response to object motion and a lagging phase in response to scene motion. The two-way repeated measure ANOVA for the phase of coherence of the low frequency pairing demonstrated no significant main effect of Coherence Stimulus Condition (p = 0.363; F = 1.148; df = 4.20, ${\eta }_{p}^{2}$= 0.18), interaction effect Coherence stimulus condition × Group (p = 0.202; F = 1.646; df = 4.20, ${\eta }_{p}^{2}$= 0.25), and between-subject effects (p = 0.415; F = 0.690; df = 1.23, ${\eta }_{p}^{2}$= 0.03). The two-way repeated measure ANOVA for the phase of coherence of the high frequency pairing demonstrated a significant main effect of Coherence Stimulus Condition (p < 0.001; F = 131.79; df = 4.20, ${\eta }_{p}^{2}$= 0.96). No other significant effects were observed for the interaction of Coherence Stimulus Condition × Group (p = 0.522; F = 0.830; df = 4.20, ${\eta }_{p}^{2}$= 0.14) and between-subject effects (p = 0.197; F = 1.76; df = 1.23, ${\eta }_{p}^{2}$= 0.25). Subsequent post-hoc analyses revealed a significant difference between the Tree Low condition and Scene Low, Scene High, and Combined (with respect to the scene) conditions (p < 0.05). A significant difference between the Combined (with respect to the tree) and Scene Low, Scene High, and Combined (scene) conditions was also observed (p < 0.05).

### Effect of visual perturbations on stepping kinematics

**Fig. 5:**
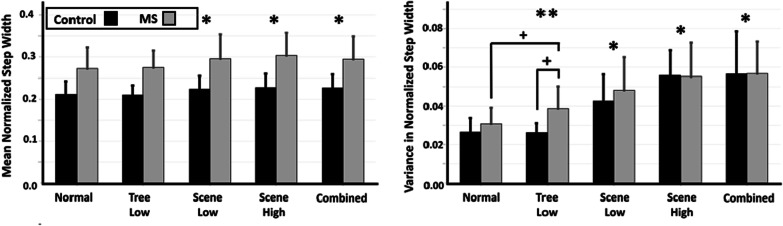
SW averages (± SD) across all participants for the control (black) and MS (grey) groups for mean normalized step width (left) and variability in normalized step width (right). A single asterisk denotes a significant (p < 0.05) main effect of condition, while a double asterisk denotes a significant (p < 0.05) interaction effect between Condition*Group, both with respect to the pair’s comparison to the ‘Normal’ condition. Subsequent post hoc analysis significance (p < 0.05) is denoted by crosses

PwMS demonstrated significant changes in SW with object perturbations that were not identified in controls, while both groups demonstrated changes in SW in response to scene perturbations. Using a repeated measures two-way ANOVA with a pairs comparison to the Normal condition, significant changes in SW were identified (Fig. 5). Step width (Fig. 5, left) illustrated significant increases in the main effect of condition from Normal in the Scene Low (p < 0.01; F = 8.89; df = 1.23, ${\eta }_{p}^{2}$= 0.28), Scene High (p < 0.01; F = 12.64; df = 1.23, ${\eta }_{p}^{2}$= 0.36), and Combined (p < 0.05; F = 7.05; df = 1.23, ${\eta }_{p}^{2}$= 0.24) conditions. Variance in step width (Fig. 5, right) illustrated significant increases in SW from Normal for the Scene Low (p < 0.001; F = 44.35; df = 1.23, ${\eta }_{p}^{2}$=0.66), Scene High (p < 0.001; F = 94.42; df = 1.23, ${\eta }_{p}^{2}$ 0.80), and Combined (p < 0.001; F = 67.54; df = 1.23, ${\eta }_{p}^{2}$= 0.75) conditions. A significant interaction effect (Condition × Group) from Normal was found in the Tree Low (p < 0.05; F = 6.99; df = 1.23, ${\eta }_{p}^{2}$= 0.23) condition, and subsequent post-hoc analysis revealed a significantly higher SW variability in MS compared to controls in the Tree Low condition (p < 0.01; t = − 3.665; df = 18.144, Cohen’s d = − 1.35). The MS group also demonstrated a significant increase in SW variability in the Tree Low condition compared to the Normal condition (p < 0.01; t = − 3.366; df = 13; Cohen’s d = − 0.79).

### Effect of visual perturbations on control of center of mass sway

**Fig. 6:**
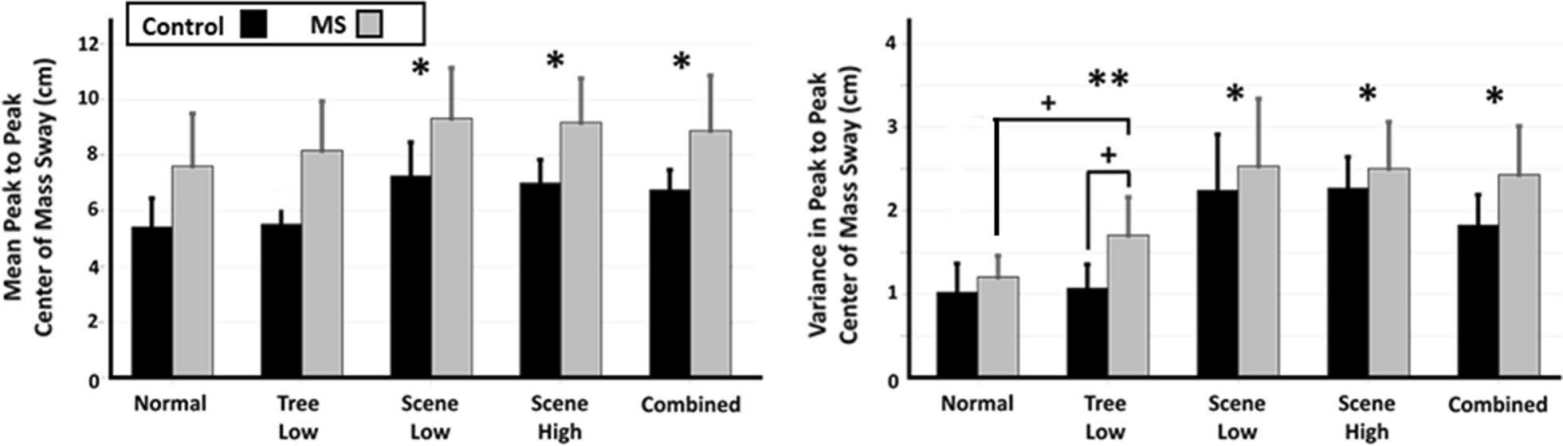
Metrics for the movement of the center of mass (± SD) across all participants for the control (black) and MS (grey) groups for mean peak-to-peak center of mass sway (cm, left) and variability in peak-to-peak center of mass sway (cm, right). A single asterisk denotes a significant main effect of condition, while a double asterisk denotes a significant interaction effect between Condition*Group, both with respect to the pair’s comparison to the Normal condition. Subsequent post hoc analysis significance is denoted by crosses. All statistical tests were considered significant at p < 0.05

PwMS demonstrated additional significant changes in control of CoM movement with object perturbations not demonstrated in controls, while both groups demonstrated changes in response to scene perturbations. Using a repeated measures two-way ANOVA with a pairs comparison to the Normal condition, significant changes in the control of the CoM sway were found with visual perturbations (Fig. 6). Peak-to-peak CoM (Fig. 6, left) significantly increased from Normal for the Scene Low (p < 0.001; F = 23.21; df = 1.23, ${\eta }_{p}^{2}$=0.50), Scene High (p < 0.001; F = 37.58; df = 1.23, ${\eta }_{p}^{2}$=0.62), and Combined (p < 0.001; F = 17.26; df = 1.23, ${\eta }_{p}^{2}$= 0.43) conditions. Variance in peak-to-peak CoM (Fig. 6, right) significantly increased from Normal in the Scene Low (p < 0.001; F = 59.64; df = 1.23, ${\eta }_{p}^{2}$= 0.72), Scene High (p < 0.001; F = 140.16; df = 1.23, ${\eta }_{p}^{2}$= 0.86), and Combined (p < 0.001; F = 78.94; df = 1.23, ${\eta }_{p}^{2}$= 0.77) conditions. A significant interaction effect (Condition × Group) from Normal was found in the Tree Low (p < 0.01; F = 10.78; df = 1.23, ${\eta }_{p}^{2}$= 0.32) condition, and subsequent post-hoc analysis revealed a significantly higher peak-to-peak CoM variability in MS compared to controls in the Tree Low condition (p < 0.001; t = − 4.22; df = 23, Cohen’s d = − 1.70). The MS group demonstrated a significant increase in peak-to-peak COM variability in the Tree Low condition compared to the Normal condition (p < 0.01; t = − 4.47; df = 13, Cohen’s d = − 1.33).

### Effect of visual perturbations on foot placement

**Fig. 7:**
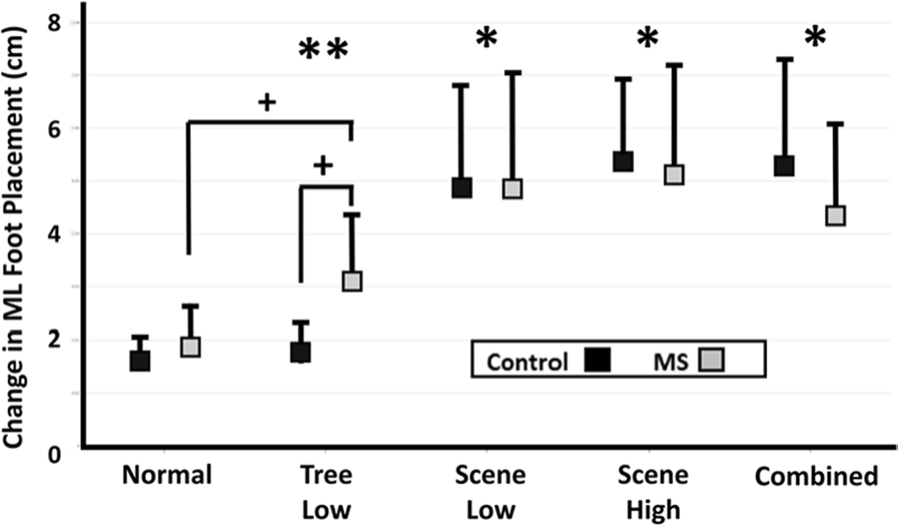
Mean change in medial–lateral foot placement (cm, ± 1 SD) across all participants for the control (black) and MS (grey) groups. A single asterisk denotes a significant main effect of condition, while a double asterisk denotes a significant interaction effect between Condition × Group, both with respect to the pairs comparison to the Normal condition. Subsequent post hoc analysis significance is denoted by crosses. Statistical tests were considered significant at p < 0.05

PwMS demonstrated significant changes in medial–lateral foot placement with object perturbations that were not present in controls, while both groups demonstrated changes in response to scene perturbations. Using a repeated measures two-way ANOVA within each group, compared to Normal walking, significant changes in medial lateral foot placement were found (Fig. 7). Change in medial–lateral foot placement illustrated significant increases from Normal in the Scene Low (p < 0.001; F = 59.12; df = 1.23, ${\eta }_{p}^{2}$= 0.72), Scene High (p < 0.001; F = 49.39; df = 1.23, ${\eta }_{p}^{2}$=0.68) and Combined (p < 0.001; F = 60.00; df = 1.23, ${\eta }_{p}^{2}$= 0.73) conditions. A significant interaction effect (Condition × Group) from Normal was found in the Tree Low (p < 0.01; F = 9.93; df = 1.23, ${\eta }_{p}^{2}$= 0.30) condition, and subsequent post-hoc analysis revealed a significantly higher change in medial–lateral foot placement in MS compared to controls in the Tree Low condition (p < 0.01; t = − 3.31; df = 13, Cohen’s d = − 1.22). The MS group demonstrated a significant increase in the change in medial–lateral foot placement in the Tree Low condition compared to the Normal condition (p < 0.01; t = − 4.42; df = 13, Cohen’s d = − 1.07). Therefore, PwMS demonstrated significant changes in medial–lateral foot placement with object perturbations that were not observed in controls, while both groups demonstrated changes in response to scene perturbations.

## Discussion

Our findings provide evidence that movements of objects within the visual field disrupt balance control in PwMS. As hypothesized, both PwMS and healthy controls altered gait in response to visual oscillations of the scene (i.e., the entire visual field). This was demonstrated by visuomotor entrainment, manifested by coherence of the medial–lateral CoM and visual field oscillation signals. In addition, we observed increased peak-to-peak CoM movement in the medial–lateral direction, increased variance of the CoM movement and increased SW with scene oscillations in both groups. However, only PwMS demonstrated evidence of instability in dynamic balance control with visual object motion, as demonstrated by increased variability in CoM sway, variability in step width, and control of foot placement. As we discuss in more detail below, we interpret these findings to suggest that PwMS likely misinterpret object movement as self-movement, causing a disruption in balance control during ambulation. This knowledge increases our understanding of potential causes of falls in MS and might be used to provide more clinically relevant rehabilitation regimens.

We observed entrainment of CoM movement with visual scene oscillations while walking on the treadmill in both PwMS and controls. Our observations are consistent with previous studies that show oscillatory movement of the CoM is altered from normal ambulation to reflect visual oscillation driving frequencies [27, 30, 31]. Processed information about the optical flow and motion parallax is typically interpreted as providing information about current heading and self-motion [27]. While visual cuing of self-motion from the visual oscillations contradicts vestibular and proprioceptive feedback, the visual system is preferentially utilized in the perception of motion [45] and corrective movements to gait were made in response to visual cues in the current study. Increased CoM sway in response to visual oscillations during ambulation indicates an increased gain in visual feedback for balance control in the elderly, who are at higher risk of falls [31]. In contrast, our study indicated that both PwMS (who are at higher risk of falls) and healthy controls react similarly to simulated self-motion via scene oscillations. Evidence of visuomotor entrainment was illustrated in both groups with high coherence values (~ 0.8) at the visual scene stimulus frequencies and increases in average peak-to-peak CoM sway.

In this study, oscillations of objects within the visual field challenged gait in PwMS. During gait, trees in the scene visually swayed left to right at a low frequency (pairing of 0.10 and 0.31 Hz). Coherence analysis of the medial lateral movement of the CoM indicated both groups changed frequency of their movement of the CoM to reflect the movement of the trees, albeit at a lower magnitude than scene oscillations. However, healthy controls did not significantly increase variability in peak-to-peak CoM sway, step width variability, or medial–lateral foot placement compared to normal walking, while increases in these variables were observed in PwMS. One explanation for the lack of response in controls could be that scene oscillations included more motion cues and therefore increased the sense of movement via optical flow and motion parallax [27], compared to diminished cues of movement presented by only tree sway. Yet, object motion and visual field movement are processed using separate mechanisms [37]. While increased cognitive load associated with tree sway might have contributed to changes in dynamic balance control in PwMS, we believe a more likely explanation for the observed response to object motion is error in differentiation of object motion from self-motion.

Previous studies in healthy adults have shown that object motion can be mistaken for self-motion due to the activation of common neurons involved in motion processing [46]. Physiologically, visual motion is interpreted and processed across multiple pathways, but most prominently through the connection from visual area 5 (V5) to the medial superior temporal area (MST). The dorsomedial region of MST (MSTd) is associated with processing self-motion, in which the neurons fire in response to contracting, expanding and translational movements within large receptive fields [47]. On the other hand, the lateroventral region of MST (MSTi) responds more strongly motion contrast between the center and periphery within smaller receptive fields and has little response to movement patterns associated with self-motion [47]. For object motion specifically, differences in brightness gradient, shape, and speed are used for motion identification [48]. This could be impaired in PwMS, as low contrast detection has been demonstrated by decreased low contrast letter acuity scores [49] and disrupted contrast perception associated can impact the perception of form from motion [39, 40]. Additionally, demyelination and lesions (reflected by reduced grey matter volume in MS [50] may impact the visual systems processing (such as in the MSTd and MSTi). These combined impairments in visual processing in PwMS could cause object motion to be interpreted as self-motion.

Perceived object and self-motion are obtained by integrating input from visual, somatosensory, and vestibular senses and the resulting perception is used in dynamic balance control. Retinal motion and extraretinal cues are compared to perceived object motion and then self-motion can be determined by comparison to efferent copies of motor commands and afferent information from vestibular and proprioceptive systems [37]. The effects of this integration process on balance have been exemplified in older adults. Thomas and colleagues reported decreased balance control while tracking object movement, which could result from challenges in estimating self-motion during object tracking [51]. Moreover, impairments in somatosensory [2] and vestibular systems [52] in MS likely lead to an increased reliance on the visual system for balance [53]. Together, these effects could cause movements within a visual scene to be mistakenly perceived as self-motion during gait, similar to way in which translations of the visual scene are perceived as changes in self-motion that lead to corrective adjustments in gait [27, 28, 30, 31]. This could suggest that PwMS are more suspectable to misinterpretation of object motion as self-motion.

Our results demonstrated that when scene oscillations and object oscillations were combined, coherence between object oscillation and CoM movement occurred in both groups. This was somewhat unexpected as partial suppression of object motion detection is produced by concurrent self-motion stimuli [54]; consequently, the observer might be expected to disregard discordant object motion cues when judging self-motion [55]. While entrainment to the object motion was reduced somewhat, the results suggest that object motion was, at least partially, interpreted as self-motion in our experiments, as it was not fully suppressed by the presence of simulated self-motion (i.e., scene oscillation). However, the phase coherence results indicate a lead response to object oscillations that was distinctly different from the lag response observed to scene oscillations. This suggests that object and scene movements were being processed differently for the control of gait. An important contributing factor could be the perspective from which motion was viewed. Visual motion interpreted as foreground (i.e., object motion) has been shown to induce a postural response in the opposite direction while motion perceived as background (i.e., scene oscillation) induces postural responses in the same direction as the movement [56]. Alternatively, it is possible that the changes in phasing may be due to the location of the scene reference point relative to the object motion. While participants were instructed to stare straight ahead to reduce effect of reference point as a confounding factor, the movement of the scene relative to the tree could have been interpreted as the reference motion, leading to the opposite sign of the phasing.

Another possible explanation for the increased gait variability seen in PwMS with object motion is that the presence of moving objects increased cognitive load in a population with decreased cognitive processing capacity. PwMS have reduced attentional focus [57] and visuospatial difficulties in adapting to complex environments [58]. Reduced information processing speed [59], visual processing [32], and object recognition [33] in MS may further impair processing of a visual scene and challenge gait stability in PwMS. Thus, object movement might affect dynamic balance control due to an impaired ability to allocate tasks in the prefrontal cortex in PwMS [36], as well as diminished cerebral recruitment with increasing cognitive demand [60]. Previously, it has been demonstrated that an increase in cognitive load can impair gait, including decreases in gait speed [34]. While the movement of objects in the visual field might increase cognitive load, coherence between CoM movement and object movement was still observed in PwMS, suggesting that object motion was incorporated into the control of gait. It is important to note that during the experiment, cognitive load was not measured and therefore further experimentation would be needed to test this possibility.

Finally, in this study there might have been a “ceiling effect”, in which visually induced changes in gait reach a saturation level as task complexity increases [61], or even a cancellation effect (object and scene movements cancel each other out due to opposite postural responses). This might explain why similar changes in gait were observed in the ‘Scene High’ condition and “Combined” conditions. The ‘Combined’ condition includes both object and scene motion, yet the resulting kinematic changes are similar to the scene motion conditions. The coherence of the CoM motion to both the object and scene frequencies suggests that both are still incorporated into the response. Including additional conditions such as a Tree High or Combined Tree High condition could have improved the interpretation of these results; however, these conditions were not included in this study due to experiment time limitations imposed to decrease the chance of motion sickness. Additionally, there were other limitations to this study. First, our sample size was relatively small (11 and 14 for controls and PwMS respectively). This sample size was chosen based on: (1) our previous work [53] investigating differences in standing balance and visual oscillations between controls and PwMS, in which sample sizes of 10 and 10, respectively, produced significant results; (2) published work [17] which reported that visual perturbations during gait produce a significant difference between controls and PwMS with a sample size of 14 and 14; and (3) despite our small sample size we obtained adequate effect sizes and relatively high power (on average > 0.85). However, we do suggest that given the nonhomogeneous nature of MS, the results may have limited applicability to cases that differed from our sample and a larger group may be needed for broader interpretation. A second limitation to the study was a significant difference between walking speed in PwMS and controls, which produced different visual flow feedback during the test. It is important to note, however, that this difference is within the range of what is considered minimal clinically different (0.1–0.2 m/s) by Bohannon et al. [65]. While the 0.16 m/s mean difference in walking speed between groups could have affected the results, no effect of gait speed was observed when included as a covariate, and previous work suggests that small changes in gait speed/visual flow produce minimal changes in response to medial/lateral visual perturbations [17]. Lastly, Expanded Disability Status Scale (EDSS) scores were not obtained for the PwMS because a trained assessor was unavailable at the times of testing. As a result, the description of the participants relies on measures of gait speed and dynamic balance.

## Conclusions

In conclusion, this study demonstrated medial lateral oscillations of a visual scene provided via a VR headset challenged gait in both healthy controls and PwMS. Increased SW, increased peak-to-peak CoM sway and increased variability in both measures confirms this conclusion. Additionally, changes in medial–lateral foot placement increased, and a high coherence between medial–lateral CoM motion and scene oscillation indicated a visuomotor effect. However, object motion within the scene, presented as sway of virtual trees, challenged dynamic balance significantly only in PwMS, as demonstrated by increased variability in step width and peak-to-peak CoM sway, as well as increased change in medial–lateral foot placement. Our interpretation of the results supports our hypothesis that PwMS are unable to properly parse object movement from self-movement, causing a disruption in postural sway during ambulation when objects move independently in the visual scene. Future studies may use these findings to foster dynamic balance control and prevent falls in PwMS by creating challenging virtual environments (such as grocery stores with object motion) within a safe laboratory setting.

## Data Availability

Deidentified datasets used and/or analyzed during the current study are available from Brian D. Schmit, PhD upon reasonable request.

## Abbreviations

PwMS: People with multiple sclerosis
MS: Multiple sclerosis
CoM: Center of mass
VR: Virtual reality
FGA: Functional gait assessment
BBS: Berg balance scale
HMD: Head-mounted display
SSQ: Simulator sickness survey
SW: Step width
MST: Medial superior temporal area
